# Depression in men with testosterone deficiency (Preliminary results of the study)

**DOI:** 10.1192/j.eurpsy.2022.813

**Published:** 2022-09-01

**Authors:** Y. Osadshiy, V. Soldatkin

**Affiliations:** 1 Rostov State Medical University, Department Of Psychiatry, Addictology And Medical Psychology, Volgograd, Russian Federation; 2 Rostov State Medical University, Department Of Psychiatry, Addictology And Medical Psychology, Rostov-on-Don, Russian Federation

**Keywords:** depression, men, testosterone, hypogonadism

## Abstract

**Introduction:**

Clinical practice in psychiatry is shifting toward personalized approach. In other words, clinicians aim to help patients based on their individual characteristics. It’s known that testosterone play a crucial role in the regulation of the emotions specially in men. The problems of hypogonadism and its possible role as an etiological factor in the development of depression in men are available in detail. But there is no solid date about the features of depression in men with testosteron defficency and theraputic approach including testosterone replacement therapy and antidepressants.

**Objectives:**

Аssessment of efficiency of different pharmacological approaches in the treatment of depression in men with testosterone deficiency

**Methods:**

The main group included 37 men with a depressive episode that arose against the background of a decrease in testosterone levels (≤12.1 nmol / L). A depressive episode was diagnosed based on the ICD-10 criteria for a depressive episode (F32). Patients were randomized into 3 treatment groups, depending on the received treatment: 1) sertraline; 2) testosterone gel; 3) sertraline + testosterone gel. The control group consisted of men (n = 40) aged 18 to 65 years, suffering from depression in accordance with the ICD-10 criteria with normal testosterone levels

**Results:**

An insufficient effectiveness of antidepressant monotherapy in relation to sexual dysfunction was found in main group, while testosterone monotherapy did not give statistically significant improvements in depression indicators.

**Conclusions:**

Combination therapy was most effective for the main symptoms and can be regarded as the most appropriate algorithm for the treatment of depression in men with low testosterone levels

**Disclosure:**

No significant relationships.

